# Demonstrating Brain-Level Interactions Between Visuospatial Attentional Demands and Working Memory Load While Driving Using Functional Near-Infrared Spectroscopy

**DOI:** 10.3389/fnhum.2018.00542

**Published:** 2019-01-23

**Authors:** Jakob Scheunemann, Anirudh Unni, Klas Ihme, Meike Jipp, Jochem W. Rieger

**Affiliations:** ^1^Department of Psychology, University of Oldenburg, Oldenburg, Germany; ^2^Department of Psychiatry and Psychotherapy, University Medical Center Hamburg-Eppendorf, Hamburg, Germany; ^3^Institute of Transportation Systems, German Aerospace Center (DLR), Braunschweig, Germany

**Keywords:** driver state assessment, mental workload, driver workload estimation, visual-motor coordination, visual attention, brain-level interactions, dual-task, fNIRS

## Abstract

Driving is a complex task concurrently drawing on multiple cognitive resources. Yet, there is a lack of studies investigating interactions at the brain-level among different driving subtasks in dual-tasking. This study investigates how visuospatial attentional demands related to increased driving difficulty interacts with different working memory load (WML) levels at the brain level. Using multichannel whole-head high density functional near-infrared spectroscopy (fNIRS) brain activation measurements, we aimed to predict driving difficulty level, both separate for each WML level and with a combined model. Participants drove for approximately 60 min on a highway with concurrent traffic in a virtual reality driving simulator. In half of the time, the course led through a construction site with reduced lane width, increasing visuospatial attentional demands. Concurrently, participants performed a modified version of the *n*-back task with five different WML levels (from 0-back up to 4-back), forcing them to continuously update, memorize, and recall the sequence of the previous ‘*n*’ speed signs and adjust their speed accordingly. Using multivariate logistic ridge regression, we were able to correctly predict driving difficulty in 75.0% of the signal samples (1.955 Hz sampling rate) across 15 participants in an out-of-sample cross-validation of classifiers trained on fNIRS data separately for each WML level. There was a significant effect of the WML level on the driving difficulty prediction accuracies [range 62.2–87.1%; χ^2^(4) = 19.9, *p* < 0.001, Kruskal–Wallis *H* test] with highest prediction rates at intermediate WML levels. On the contrary, training one classifier on fNIRS data across all WML levels severely degraded prediction performance (mean accuracy of 46.8%). Activation changes in the bilateral dorsal frontal (putative BA46), bilateral inferior parietal (putative BA39), and left superior parietal (putative BA7) areas were most predictive to increased driving difficulty. These discriminative patterns diminished at higher WML levels indicating that visuospatial attentional demands and WML involve interacting underlying brain processes. The changing pattern of driving difficulty related brain areas across WML levels could indicate potential changes in the multitasking strategy with level of WML demand, in line with the multiple resource theory.

## Introduction

Driving is a complex task, composed of multiple subtasks where different cognitive demands are concurrently imposed on the driver. For instance, one needs to be attentive toward unforeseen events, integrate information from within and outside the vehicle, and control the vehicle to keep it on the lane. All those tasks require cognitive resources of limited capacity (Wickens et al., 2008). Some of these tasks could possibly draw from the same shared resources, leading to a potential interaction between different subtasks.

Working memory plays an important role while driving since the driver has to continuously integrate and dynamically update information from internal and external traffic environments (De Waard, 1996; da Silva, 2014). For example, Wood et al. (2016) have associated increased working memory capacity with better ability to control visual attention while being less distracted in different driving tasks. Further, certain driving situations are associated with increased working memory demands, e.g., left turns at intersections (Guerrier et al., 1999) or driving within a dense city environment (Patten et al., 2006) as they require integration of more items into trajectory planning. Yet, working memory is a capacity-limited system (Baddeley, 2003; Cowan, 2010) and working memory overload deteriorates driving performance (Lavie, 2010). For example, it has been shown that increasing working memory load (WML) via a secondary task decreases driving performance on the lane change task (Ross et al., 2018). Interestingly, this effect was larger for people with less working memory capacity.

Besides working memory, driving requires visuospatial attention and visuomotor control (Vingerhoets and Stroobant, 1999; Lust et al., 2011; Benedetto et al., 2013). Visual attention is demanded because the driver needs to simultaneously integrate central and peripheral vision within a rapidly changing moving environment, while monitoring for unexpected critical events (Owsley and McGwin, 2010). Under decreased vision, more resources are allocated to lane keeping (Gao and Zhang, 2016). More specifically, Brooks et al. (2018) could link a decrement in driving performance in a lane-keeping task to increased peristimulus alpha activity, an indication for poor visuospatial attention. Further, when participants drove in a narrow road condition as compared to the ordinary driving task with normal lane widths, fNIRS measured increased activation in the prefrontal areas (Shimizu et al., 2009). This supports other findings showing that driving in narrowed lanes is more demanding (De Waard et al., 1995; Liu et al., 2016a) and associated with performance loss (Rosey and Auberlet, 2012). Thus, narrowed lanes seem to increase visuospatial attention load necessary for controlling the vehicle safely.

In driving, different task demands interact with each other (Borghini et al., 2014; Matthews et al., 2015). On the behavioral level, there are various studies that have investigated the effect of cognitive load on driving performance. For example, a majority of the studies suggest that cognitive load actually improves driving performance indicated by improved lane keeping (He and McCarley, 2011; Cooper et al., 2013; for review see Engström et al., 2017). Yet, for studies in which driving difficulty was increased by exposing the car to crosswinds, an additional cognitive load task led to an improvement in lateral control in one study (He et al., 2014), but a drop in another study (Medeiros-Ward et al., 2014).

The interaction of workload and driving performance on the neural level was studied by Wang et al. (2018). In their driving study, using electroencephalography (EEG), car drifts were induced requiring the participant to make lane-keeping adjustments. Additionally, a mathematical calculation task was presented either right before, right after or simultaneously to the induced car drifts. Theta and alpha oscillations in frontal, parietal and occipital areas in the different dual-task conditions were compared to oscillations in single task conditions. While over-additive activation in the frontal theta oscillations were found for the simultaneous condition, all other location-band combinations revealed either additive or under-additive activation in dual-tasking. Vossen et al. (2016) studied the effect of WML on the temporal neural markers for visuospatial attention. Participants performed worse in a visuospatial attention task, in which participants had to react to specific cued visual stimuli in a traffic scenery, when they had to complete an additional verbal memory rehearsal task simultaneously. A further analysis of evoked response potentials (ERPs) from EEG showed that in the high WML conditions, there was a reduction and delay of neural markers only in the early stages of the visuospatial task associated with the initiation of spatial orienting. On the contrary, later stages of the visuospatial task responsible for retaining attentional focus and target selection revealed no differences in the high WML conditions.

The effect of an additional task on the primary driving task was also studied with functional magnetic resonance imaging (fMRI). In a driving simulator study, Just et al. (2008) found a decrease in parietal activation associated with spatial attention in normal driving when participants performed an additional listening comprehension task. As spatial attention and listening comprehension draw resources mostly from non-overlapping cortical areas, the authors interpret the “diversion of attention as reflecting capacity limit on the amount of attention or resources that can be distributed across the two tasks” (p. 76). Similarly, in a more recent fMRI-driving simulator study, Choi et al. (2017) found a decrease in activation in the parietal areas and an increase in activation in the inferior frontal gyrus and the superior temporal gyrus associated with an additional listening comprehension task while driving. These results illustrate the complex interaction of how an additional task alters the neural activation associated with the primary driving task.

In a cognitive approach on dual-tasking, Wickens (2008) defined resources in his multiple resource theory of attention along four dimensions, namely stages of processing, codes of processing, modalities, and visual channels. The model assumes an interference in dual-tasking when tasks compete for the same resources. For each task, a computational model codes the amount of resources needed for each dimension. For any dimension, if all tasks combined require more resources than what is available, the model predicts interference and performance loss (Wickens, 2002). In an earlier study, the model was implemented to predict driving performance along nine different dual-task combinations consisting of different driving conditions (e.g., urban vs. rural routes) and additional different secondary tasks (e.g., visual vs. auditory backward reading of numbers; Horrey and Wickens, 2003). Performance loss in dual-tasking was successfully predicted by the model for latency of the secondary task and response times to critical road hazards.

An important aspect of the multiple resource theory is executive control, which describes the allocation of resources between tasks. Especially in situations of high dual-task demands, resources might be drawn away from a less prioritized task toward a task with higher priority. Hence, the amount of resources allocated to a subtask depends on the demands of the other subtask, in particular when the other subtask is prioritized. However, how these interactions happen on the brain level in real world tasks is largely unknown. Therefore, in this study, we aimed to investigate at the brain level, how different task demands in one cognitive domain affect the resource allocation for another cognitive domain, by comparing the specificity of predictive brain activation patterns across various dual-tasking scenarios. Specifically, we sought to explore how the assessment of visuospatial attentional driving demands from functional near-infrared spectroscopy (fNIRS) measurements depends on different WML levels.

Functional near-infrared spectroscopy has recently become popular in driving research as a measure of brain activity because it provides brain activations measures with reasonable anatomical and temporal resolution in relatively unconstrained applied settings (Liu et al., 2016b; Sibi et al., 2016). FNIRS uses near-infrared light to measure local concentration changes of deoxygenated hemoglobin (HbR) and oxygenated hemoglobin (HbO) from cortical brain areas which are seen as correlates of functional brain activity (Villringer et al., 1993; Sassaroli and Fantini, 2004). In comparison to HbO, HbR signals are considered to be less influenced by systemic physiological artifacts like cardiac pulsation, respiration, or Mayer wave fluctuations than HbO (Obrig et al., 2000; Zhang et al., 2005, 2009; Huppert et al., 2009; Suzuki, 2017). Other studies additionally reported that HbR tends to correlate stronger with blood oxygenation level dependent (BOLD) response than HbO (MacIntosh et al., 2003; Huppert et al., 2006; Schroeter et al., 2006; Foy et al., 2016).

In comparison to fMRI, fNIRS has lower spatial (Cui et al., 2011; Mehta and Parasuraman, 2013; Pinti et al., 2018), but better temporal resolution (Huppert et al., 2006). Compared to EEG, fNIRS has lower temporal (Naseer and Hong, 2015), yet better spatial resolution (Scholkmann et al., 2014). Due to its robustness against motion artifacts and external electrical noise, fNIRS is suitable for applied settings (Masataka et al., 2015; Balardin et al., 2017) and has been used in actual driving (Yoshino et al., 2013a,b). FNIRS has shown to be sensitive toward changes in mental workload in the applied fields of simulated flight operation (Ayaz et al., 2012; Durantin et al., 2014), simulated urban rail driving (Li et al., 2018), as well as simulated (Unni et al., 2017; Xu et al., 2017) and actual car driving (Ahn Son et al., 2018). Further, fNIRS could detect elevated visual attention in curve driving, as indicated by increased activity in right premotor cortex, right frontal eye field, and bilateral prefrontal cortex (Oka et al., 2015). Thus, fNIRS is applicable in applied driving settings while providing independent measures of activity in functionally specific brain areas.

In this study [some data has already been published in Unni et al. (2017)], we used fNIRS brain activation measurements obtained during driving to predict two types of cognitive demands: visuospatial attentional demands and working memory demands, both modulated simultaneously. To manipulate visuospatial attentional driving difficulty, participants drove in a 360° Virtual-Reality (VR) driving simulator, half of the time through a construction site with a reduced lane width. At the same time, participants had to perform the primary driving task, which was a working memory speed regulation task (Unni et al., 2017) with five different WML levels. Recording almost whole-head fNIRS brain activation measurements, we aimed at predicting driving difficulty (i.e., driving outside and within construction sites with narrower lane widths) as a measure for visuospatial attentional demands. One of our central questions was whether it is possible to predict driving difficulty or whether task interactions between visuospatial attentional demands and WML levels at the brain level render this impossible. More precisely, we calculated decoding models for the prediction of driving difficulty from almost whole-head fNIRS for each WML level separately and a model which combined fNIRS data over all WML levels. A model with good prediction accuracy for driving difficulty can be interpreted such that there exist distinct neural correlates associated with increased driving difficulty. If there was no interaction between WML and visuospatial attention, a decoding model which combined fNIRS data over all WML levels would perform similarly well in predicting driving difficulty as using a decoding model for each WML level separately. However, if there was an interaction between visuospatial attentional demands and working memory demands, activation patterns associated with increased driving difficulty would differ over WML levels leading to better prediction accuracy for the separate models. Hence, the comparison of prediction accuracies of the different decoding models characterizes the interaction between the visuospatial attention with working memory processing at the brain level. This is relevant for the development of brain-based driver assistive systems as well as for understanding the nature of the multitasking interactions at the brain level.

## Materials and Methods

The experiment was implemented in a driving simulator where participants drove on a highway with varying concurrent traffic. Participants performed a driving task in a two factorial within participant design with factors driving difficulty manipulated by visuospatial attentional demands (two levels: non-construction and construction) and WML (five levels: 0–4 back). The driving difficulty was manipulated via changes of lane width and for WML manipulation, participants performed a digit-span *n*-back speed regulation task. The details of the tasks are provided below.

### Participants

Nineteen volunteers (17 males) aged 19–32 years (Mean ± *SD* = 25.2 ± 3.7) participated in the experiment. All participants possessed a valid German driving license at the time of the experiment. Participants gave informed consent prior to the experiment and received a financial reimbursement of 10 € per hour. The experiment was conducted according to the guidelines of the German Aerospace Center and was approved by the Ethics Committee of the Carl von Ossietzky University, Oldenburg.

### Experimental Set-Up

The experiment was set up in a VR-lab at the German Aerospace Research Center allowing a 360° full view (Fischer et al., 2014). During the experiment, participants were operating a realistic vehicle mock-up equipped with common throttle, brake pedal, steering wheel, and indicators. Participants drove on a simulated, slightly curvy highway (64 km in total; developed on the platform Virtual Test Drive, Vires Simulationstechnologie, Bad Aibling, Germany) with varying concurring traffic. There were 15 vehicles set randomly in an area with a radius of 1000 m around the ego vehicle. Of those vehicles, 60% followed the direction of the ego vehicle; 35% were in the front, 35% in the back, 15% to the left and 15% to the right of the ego vehicle; and 45% were trucks, other 55% were cars.

While driving, fNIRS brain activation measurements were recorded from almost whole-head at a sampling frequency of 1.955 Hertz (Hz) from thirty-two optical emitters and detectors using two NIRScout systems (NIRx Medical Technologies, LLC, United States) in tandem mode. The system uses two wavelengths of 760 and 850 nm to calculate the relative concentration changes of HbO and HbR. We defined 78 fNIRS channels (emitter-detector combinations) in total with an average channel distance of about 3.5 cm. The exact channel locations are provided in Unni et al. (2017). Along with fNIRS data, steering wheel position and driving speed was also recorded at a sampling frequency of 50 Hz.

#### Visuospatial Attention Manipulation

We manipulated the visuospatial attention demands for the driving task throughout the highway. For about half of the time, participants were driving within a construction site (labeled as *construction*). During the other half of the drive, participants were driving on a normal road without the construction site (labeled as *non-construction*).

The main differences between those two conditions were the number of available lanes and their widths. In the non-construction condition, there were three lanes available with a total width of 10.75 m, consisting of two lanes with a width of 3.5 m (left and center lane) and a slightly wider right lane with a width of 3.75 m. Driving in the construction site was more difficult where only two lanes were available. The widths of the lanes were also reduced along the construction sites with the left and right lanes having a width of 2.5 and 3.5 m, respectively, resulting in a total width of 6 m.

Further, the highway resembled the typical design of German highways. In the non-construction site, there were solid markings in white on the left and right of the road with dashed lines between the lanes. As typical for German highways, pylons marked the beginning and end of the construction sites and yellow markings highlighted the new lanes. The positions and design of the speed signs remained the same.

**FIGURE 1:**
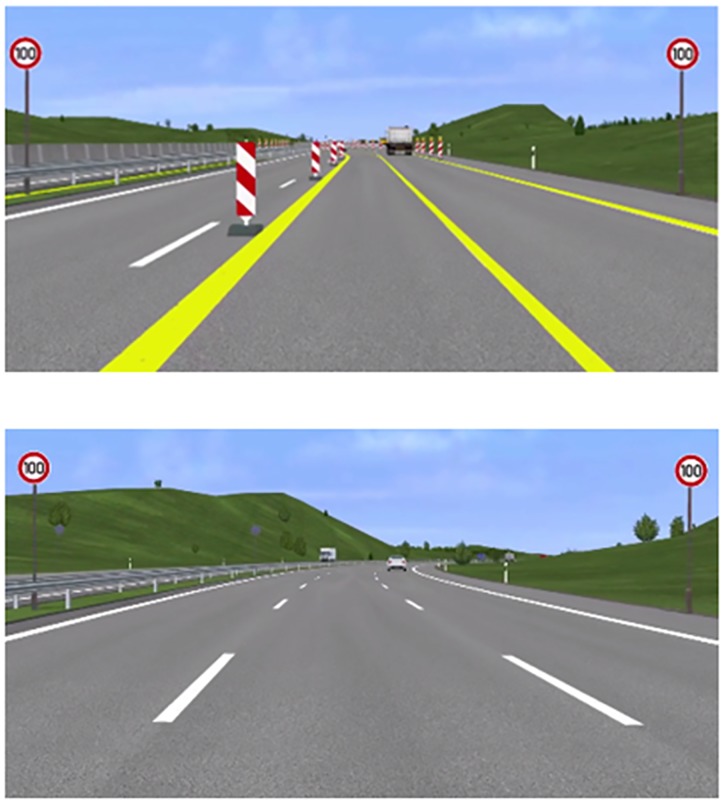
Screenshots from the experimental paradigm. **Top**: a scene from the construction condition with two lanes of reduced width. **Bottom**: a scene from the non-construction condition with three lanes and normal lane width.

Screenshots from the experimental paradigm for both conditions can be seen in Figure 1. In both conditions, participants had to avoid collisions with other vehicles in ongoing traffic and overtake when it was deemed necessary to drive at the correct speed. Speed signs and WML levels varied at the same rate over both levels of driving difficulty.

#### Working Memory Load Manipulation

**FIGURE 2:**
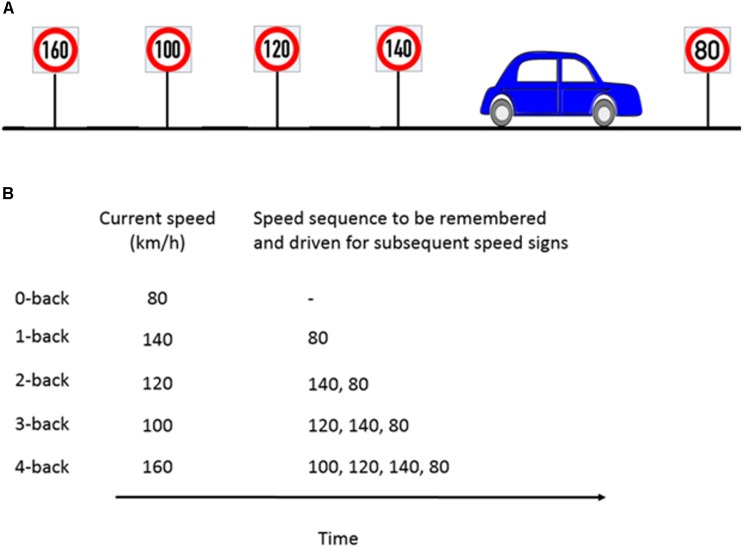
Example of the *n*-back experimental paradigm to manipulate cognitive workload. **(A)** Consider a scenario where the participant is about to pass the 80 km/h speed sign and the previous four speed signs were as shown in the schematic. **(B)** For the corresponding *n*-back task, participants had to memorize the last *n* speed signs and drive at the *n*-th speed sign which occurred previously. For example, at 1-back, the participant’s target speed is the previous sign (140 km/h) and has to keep the current speed sign in memory (80 km/h). Figure taken from Unni et al., 2017.

The *n*-back task is considered to be a benchmark for WML manipulation in neurocognitive psychology (Kirchner, 1958). In a classical *n*-back task, a series of numbers, letters, or other stimuli are presented. Participants then have to compare the current stimulus with the stimulus *n* steps back and give a response whenever they are the same. We modified the classical *n*-back task to be applicable in the driving scenario by using speed signs as stimuli. Participants had to adjust their speed to the speed sign they passed *n* speed signs before. For a successful performance, it was necessary that participants continuously update, memorize and recall the previous *n* speed signs. Our experiment consisted of five different workload levels from *n* = 0 (adjusting the speed to the current speed sign) to *n* = 4 (adjusting the speed to the 4th previous speed sign). The task is illustrated in Figure 2. A detailed explanation of the WML speed regulation task can be found in Unni et al. (2017).

Participants had a 6 s window (3 s both before and after passing the sign) to adjust their speed to the target speed. A deviation up to ±5 km/h from the target speed was judged as correct. Whenever the deviation was more than ±5 km/h, a warning message ‘Please pay attention to your speed’ was displayed on the screen. This was done to motivate the participant to drive at the correct speed. This message appeared on the screen until the participant drove within the correct speed range. For every new *n*-back task, participants were instructed to stay at the speed of the first sign until they passed ‘*n*’ successive speed signs before they could begin with the *n*-back task. There were nine different speed signs (60–140 km/h in steps of 10 km/h) presented in random order to avoid sequencing effects. At the beginning of a new *n*-back condition, participants were informed via a message displayed for 5 s on the VR-screen about the next *n*-back level to be accomplished.

#### Experimental Procedure

The participants started with a 20 min training session where they drove each of the five different *n*-back levels twice. Then, the main experiment started, which lasted about 60 min with a break in the middle. In total, the participants performed each 3 min long *n*-back level four times, twice in each of the *construction* and *non-construction* conditions. The speed signs were distributed such that the participants passed a new speed sign roughly every 20 s with some temporal jitter. The construction and non-construction sites were alternating with every change in *n*-back level. The order of the *n*-back levels was pseudorandomized in such a way that the same *n*-back level was never driven twice in a row and each *n*-back level was performed twice in the construction and the non-construction conditions respectively. Also, the sequence of *n*-back levels repeated itself in reversed order after the break to avoid sequencing effects.

### Data Analysis

#### Driving Behavior

To determine the effect of increasing WML levels, we calculated error rates in the speed regulation task. As a measure of performance in the working memory task, we calcultated the percentage of time segments in which the participant did not reach the target speed (<90% driving within the tolerance interval around the target speed). In line with the analysis for the fNIRS data described below, we have excluded those time segments (∼8% of time segments over all participants) from the other analysis of driving behavior.

In order to check whether driving through the construction site was associated with changes in driving performance, we analyzed the steering reversal rate. Steering reversal rate was defined as the number of times the participant crossed the centered position of the wheel. Steering reversal rate usually increases with increased driving difficulty, as more corrections to the steering wheel position are required (Macdonald and Hoffmann, 1980). As a measure for increased driving difficulty, we calculated the difference in steering reversal rate between driving in the construction and non-construction condition for each *n*-back level.

Due to a problem in data recording in one participant, driving behavior is presented for only 14 participants.

#### Working Memory Capacity

To ensure that all participants had comparable levels of working memory capacity, they first performed the memory updating task from the working memory capacity test battery by Lewandowsky et al. (2010). In this test, participants had to remember a set of digits which they had to update continuously through a series of simple arithmetic operations (single digit addition and subtraction). For every correct trial, participants received 1 point. The average total score was 38.4 (*SD* = 10.7) out of a maximum possible score of 60. One participant was excluded from the data analysis, because of a score more than two standard deviations below the mean.

#### FNIRS Data Processing

We used the nirsLAB analysis package (Xu et al., 2014) for fNIRS pre-processing. Physiological artifacts (heartbeat, respiration, and Mayer waves) were reduced with a low-pass filter (finite impulse response with least-square error minimization) with a cut-off frequency of 0.1 Hz. We used the Gratzer Spectrum to obtain the molar extinction coefficients of HbO and HbR corresponding to wavelengths of 760 and 850 nm, respectively (Prahl et al., 1999). The corresponding molar extinction coefficients are €_760_ = [1486.59 3843.71] and €_850_ = [2526.39 1798.64] M^-1∗^cm^-1^ (nirsLAB, NIRx Medical Technologies). The differential path length factor takes into account the increased distance the light path travels from the emitter to the detector because of scattering and absorption effects. The differential path length factors for HbO and HbR were 7.25 and 6.38, respectively (Essenpreis et al., 1993). The relative concentration changes in hemoglobin (mmol/l) were calculated via the modified Beer–Lambert’s law (Sassaroli and Fantini, 2004). For the modified Beer–Lambert’s law calculation, the exact source-detector distance for each NIRS channel was computed by nirsLAB according to the corresponding distances between emitter and detector pairs on the NIRS cap.

We computed a channel-wise coefficient of variation (CV) which is a measure for the signal-to-noise ratio (SNR) from the unfiltered raw data. CV is calculated as the ratio of the standard deviation and the mean of each NIRS channel over the entire duration of the experiment (Schmitz et al., 2005; Schneider et al., 2011). All channels with a CV greater than 20% were excluded from further analysis. On average, 64 channels per participant were included in the analysis (*SD* = 7). For the following fNIRS analysis, we have used the HbR signal.

In the fNIRS analysis, we excluded all consecutive time segments between two successive speed signs (∼20 s) in which the participant didn’t reach the target speed (∼8% of time segments over all participants). This was done because we were not sure whether the participant was continuing to focus on the working memory task in those time segments or whether he or she had already given up at an earlier stage due to the inability to focus on the task due to cognitive overload. This is important, as disengagement from difficult tasks reduces the actual cognitive load and affects interpretability of results since workload would be significantly lower than what would be expected on basis of objective task requirements (Victor et al., 2005; Mehler et al., 2012).

A common method to increase the SNR is the application of a Principal Component Analysis (PCA) on the pre-processed fNIRS data (Virtanen et al., 2009). In a PCA, the fNIRS data is transformed to a new set of variables called ‘principal components’ (PCs) that are linearly uncorrelated and ordered according to the amount of variance explained in the data. It is presumed that motion artifacts contribute more to the variance than the neurophysiological signals and hence the first PC will mostly explain variance dominated by motion artifacts. Therefore, in order to remove motion artifacts, we deleted the first PC, which has shown to be a successful procedure in motion artifact reduction of fNIRS data (Cooper et al., 2012; Brigadoi et al., 2014). Besides motion artifacts, fNIRS data contains noise, for example random instrumental white noise. As we can assume this noise to have a Gaussian distribution, all PCs will contain noise of the same Gaussian distribution. As all PCs contain the same noise variance, first PCs, which explain most of the variance will have a better SNR than later PCs, which explain little variance but will be dominated by the same noise variance and therefore have a worse SNR. That is why we retained only PCs with high exploratory value before transforming the PCs back into the time-series fNIRS data. Based on the recommendation by Jolliffe (1972) on the Kaiser’s rule (Kaiser, 1958), all components with eigenvalues larger than 0.7 were kept. With the procedure of deleting the first PC and all other PCs with an eigenvalue smaller than 0.7, 7.09 PCs (*SD* = 2.07) were retained on average over all 15 participants. As detailed in the section below, the PCs are calculated on training data in a cross-validation scheme. These retained PCs were then transformed back to the original space resulting in a less noisy time-series fNIRS data.

#### Multivariate Cross-Validated Prediction of Driving Difficulty

Our goal was to predict the driving difficulty, i.e., whether the participant was in the construction or non-construction condition. First, we calculated binary multivariate logistic ridge regression models (Hastie et al., 2009) for the prediction of driving difficulty from fNIRS data for each WML level, i.e., we calculated separate models for each of the five *n*-back levels for each participant. Second, we calculated one binary multivariate logistic ridge regression model to predict driving difficulty from fNIRS data combined over all WML levels for each participant. Both models used time-resolved fNIRS HbR pre-processed data from all the good channels at each timepoint (sampling frequency 1.955 Hz) as one signal sample. From each signal sample, channel-wise weights were used for the model, which were computed using the Glmnet toolbox (Qian et al., 2013). The output of the logistic regression model can be interpreted as a class probability. Consequently, we computed a model output for each signal sample. All samples with a model output of *p* ≥ 0.5 were assigned to the class construction. This allowed us to calculate the rates at which the model correctly classified different conditions.

In this study, we report model accuracy, which indicates the proportion of correctly classified samples as either construction or non-construction. The accuracy was calculated as follows:

$$Accuracy(\%)=\frac{{TP}_{c}+{TP}_{nc}}{{TP}_{c}+{TP}_{nc}+{FP}_{c}+{FP}_{nc}}*100$$

Here, the *TP* refers to the true positives (number of samples correctly classified) and *FP* refers to the false positives (number of samples incorrectly classified) for the two conditions denoted by *c* for construction and *nc* for non-construction.

While classification accuracy is an intuitive concept to evaluate the performance of a model, it can be biased, e.g., by uneven data sets. In contrast, precision and recall are advantageous performance measures, insensitive to training set size differences (Rieger et al., 2008). Precision provides information about how precise the model is in assigning a particular sample to the respective empirical class (‘*construction*’ or ‘*non-construction*’). On the other hand, recall is the proportion of samples belonging to a particular class (‘*construction*’ or ‘*non-construction*’) which were also assigned to the same class by the model. Here, we report the *F*1-scores which are a harmonic average of the precision and recall measures. A *F*1-score of 1 indicates perfect precision and recall (Shalev-Shwartz and Ben-David, 2014). The *F*1-score for the construction condition was calculated as follows:

$$F1-score=\frac{{2*TP}_{c}}{{2*TP}_{c}+{FP}_{c}+{FP}_{nc}}$$

In order to test the generalization of the logistic ridge regression model to new data and to avoid overfitting, an out-of-sample nested cross-validation procedure as suggested by Hastie et al. (2009) was used for model training and testing. The outer loop implemented a five-fold cross-validation where the pre-processed fNIRS time-series data was split into five consecutive blocks. In each fold, a different set of four blocks was used as training set to train the model while the left-out block was used to test the generalization of the model. In addition, an inner five-fold cross-validation loop was implemented on the training set where we first performed the PCA of the fNIRS time-series data to reduce noise, after which it was transformed back from PC-space to the original time-series space. Using the Glmnet toolbox (Qian et al., 2013), channel-wise weights for the logistic regression model were found, for which the λ regularization parameter was optimized internally by Glmnet in the training phase. The cross-validation procedure avoids overfitting of the data to the model and provides an estimate of how well a decoding approach would predict new data in an online analysis (Reichert et al., 2014).

#### Univariate Correlation Analysis

Interpreting the channel weights as indicators for brain areas involved with the experimental condition can be difficult as they result from a multivariate model and each weight can only be interpreted in the context of the whole model (Reichert et al., 2014; Weichwald et al., 2015; Holdgraf et al., 2017). To achieve better interpretability, we additionally fitted channel-wise, univariate logistic regression models of the fNIRS HbR data on the driving difficulty for each participant for the separate models. The fNIRS data was the same preprocessed data that was used for the multivariate analysis. To reduce noise and movement artifacts, we used a PCA the same way as for the multivariate analysis. We performed a PCA for each condition and participant, deleted the first and all PCs with an eigenvalue smaller than 0.7 and then transformed it back from PC-space to the original time-series space. To determine model fit, we used the method suggested by Tjur (2009), to calculate *R*^2^ as measure of the predictivity of a channel (*R*^2^*_uvr_*). The *Tjur R*^2^ varies between 0 (no predictivity) and 1 (perfect predictivity).

We created averaged predictivity maps across all participants (*Tjur R*^2^*_avg_*) for each fNIRS channel, illustrating the differences in brain activation between construction and non-construction site driving, separately for each *n*-back level. Those averages were calculated by weighting the single-subject’s univariate coefficient of determination (*R*^2^*_uvr_*) with prediction accuracy from the multivariate regression analysis:

$$Tjur\,\;R_{avg}^{2}(i)=\frac{\overset{i,n}{\underset{i,n=1}{\Sigma }}R_{uvr}^{2}(i)*Accuracy\left(n\right)}{\overset{n}{\underset{1}{\Sigma }}Accuracy\left(n\right)}$$

## Results

### Participants

Four participants were excluded from the analysis, three of them due to a large number (>50%) of noisy fNIRS channels and one due to low performance in the working memory capacity test. Thus, data from fifteen participants, all males, aged 19–32 years (Mean ± *SD* = 25.6 ± 3.96) are included in the following analysis.

### Driving Behavior

#### Steering Reversal Rate

Across all *n*-back levels, the steering reversal rate was higher in the construction condition than in the non-construction condition, indicating that the construction site increased driving difficulty (see Table 1). Additionally, this difference increased for higher *n*-back levels, with exception of the 3-back, indicating that driving difficulty increased with increasing WML levels (*r* = 0.65, *p* < 0.001). This is also supported by a two-factor analysis of variance (ANOVA) with the factors driving difficulty and WML level. For steering reversal rate we observed main effects for both driving difficulty [*F*(1,130) = 146.87, *p* < 0.001] and WML level [*F*(4,130) = 19.08, *p* < 0.001], as well as a significant interaction effect [*F*(4,130) = 10.49, *p* < 0.001]. For additional analysis on lane deviation (see Supplementary Table S1).

**Table 1:** Steering” specified in Tables 1, 2. Reversal Rate in Hertz.

	0-back	1-back	2-back	3-back	4-back	Mean
Construction	0.012	0.012	0.017	0.013	0.018	0.014
Non-construction	0.011	0.008	0.012	0.009	0.010	0.010
t_construction-non-construction_	*t*(13) = 2.027	*t*(13) = 8.123	*t*(13) = 9.817	*t*(13) = 15.571	*t*(13) = 11.445	*t*(13) = 13.821
Significance test	*p* = 0.064	*p* < 0.001	*p* < 0.001	*p* < 0.001	*p* < 0.001	*p* < 0.001
Bonferroni corrected *p*^∗^-value	*p*^∗^ = 0.318	*p^∗^*< 0.001	*p^∗^*< 0.001	*p^∗^*< 0.001	*p^∗^*< 0.001	*p^∗^*< 0.001


#### Error Rates in WML Speed Regulation Task

**FIGURE 3:**
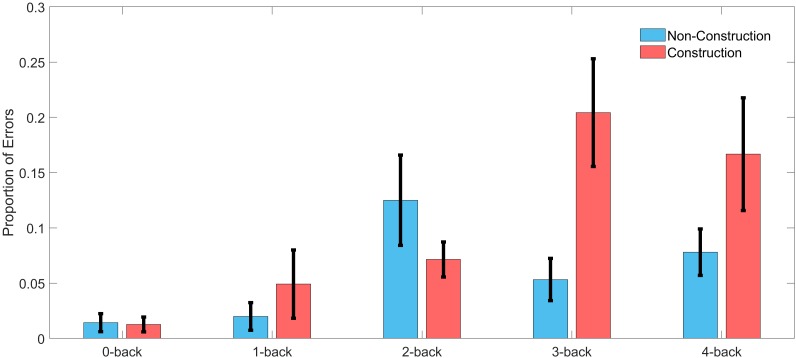
Error rate in the speed regulation task for driving in construction and non-construction condition for each *n*-back level across all participants. Black lines indicate the standard error of the mean (*n* = 15).

We calculated the error rates (percentage of target speeds the participants failed to reach) in the WML speed regulation task in the construction and non-construction condition. A two-factor ANOVA with the factors driving difficulty and WML level revealed main effects of error rates for both driving difficulty [*F*(1,130) = 5.12, *p* = 0.03] and WML level [*F*(4,130) = 6.16, *p* < 0.001], as well as a significant interaction effect [*F*(4,130) = 3.54, *p* < 0.01]. Figure 3 shows that for all *n*-back levels except for 2-back driving in the construction site was accompanied by more errors in the working memory speed regulation task as compared to driving in the non-construction site. This was especially true for the 3-back and 4-back levels (see Table 2). The reduced meory performance suggests that increased recruitment of cognitive resources required to meet increasing visuospatial attention demands for the lane-keeping task interacts with cognitive resource recruitment in the working memory task.

**Table 2:** Differences in errors between driving difficulty conditions (construction–non-construction) calculated via paired-sample *t*-test and the effect size Cohen’s *d*.

	0-back	1-back	2-back	3-back	4-back	Mean
Construction	0.01	0.05	0.07	0.21	0.17	0.10
Non-construction	0.01	0.02	0.13	0.05	0.08	0.06
Differences between driving difficulty condition	*t*(13) = -0.195 *p* = 0.849	*t*(13) = 1.011 *p* = 0.331	*t*(13) = -1.158 *p* = 0.268	*t*(13) = 3.014 *p* = 0.010	*t*(13) = 2.189 *p* = 0.046	*t*(13) = 3.198 *p* = 0.007
Cohen’s *d*	-0.06	0.52	-0.46	1.10	0.76	0.85
Bonferroni corrected *p*^∗^-value	*p^∗^* = 1.00	*p^∗^* = 1.00	*p^∗^* = 1.00	*p^∗^* = 0.050	*p^∗^* = 0.230	*p^∗^* = 0.035


### FNIRS Results

#### Prediction of Driving Difficulty

**FIGURE 4:**
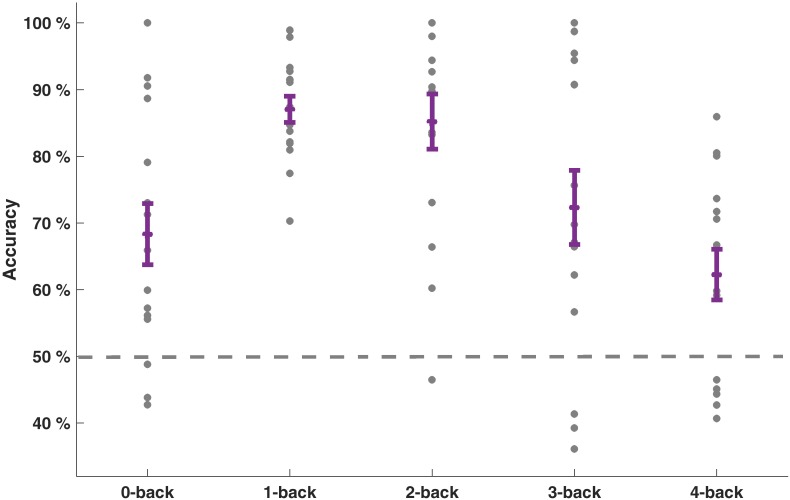
Prediction accuracies of driving difficulty for the models separate for each WML level. Individual accuracy score is indicated as dots. Mean accuracy per WML level and its standard error of the mean are depicted in purple. Dashed line at 50% indicates the theoretical guessing level.

Our goal was to classify the driving difficulty from multivariate logistic ridge regression using pre-processed fNIRS signal samples (sampling frequency 1.955 Hz) in a cross-validation scheme with five equally sized blocks to avoid class size bias. We first calculated separate models for each WML level and each participant. With this procedure, we predicted driving difficulty correctly in 75.0% of the signal samples on average over WML levels and participants. The mean *F*1-score was 0.70. The similar scores between *F*1-score and accuracy suggest that the model was not biased to a single class. There was a significant effect of the WML level on the prediction of driving difficulty as indicated by the rank-based non-parametric Kruskal–Wallis H test for both model accuracy [range: 62.2–87.1%: χ^2^(4) = 19.91, *p* < 0.001] and *F*1-scores [*range*: 0.57–0.86; χ^2^(4) = 15.46, *p* < 0.01]. Predictions were better for intermediate WML levels (1-back and 2-back) as illustrated in Figure 4 for model accuracy and Table 3 for *F*1-scores. This pattern of prediction accuracy holds for most individual participants: In 12 out of 15 participants, best model performance *F*1-scores were achieved for either 1-back or 2-back.

**Table 3:** *F*1-scores of each classifier for predicting driving difficulty and means across participants and *n*-back levels (individual maxima bold).

Participant	0-back	1-back	2-back	3-back	4-back	Mean
01	0.23	**0.77**	0.09	0.32	0.70	0.42
02	0.70	0.80	**0.88**	0.23	0.72	0.67
03	0.62	0.85	**0.98**	0.91	0.54	0.78
04	0.89	**0.98**	0.47	0.90	0.66	0.78
05	0.55	0.93	0.90	**1.00**	0.21	0.72
06	0.90	0.86	**0.88**	0.71	0.84	0.84
07	0.44	0.78	**0.94**	0.48	0.67	0.66
08	0.42	0.99	**1.00**	0.74	0.31	0.69
09	0.77	0.68	0.89	**0.94**	0.52	0.76
10	0.67	0.87	**1.00**	0.23	0.84	0.72
11	0.50	0.82	**1.00**	0.99	0.33	0.73
12	0.73	0.88	**1.00**	0.67	0.56	0.77
13	**1.00**	0.93	0.16	0.94	0.77	0.76
14	0.24	**0.91**	0.77	0.27	0.44	0.53
15	0.89	**0.91**	0.57	0.31	0.40	0.61
**Mean**	**0.64**	**0.86**	**0.77**	**0.64**	**0.57**	**0.70**


**FIGURE 5:**
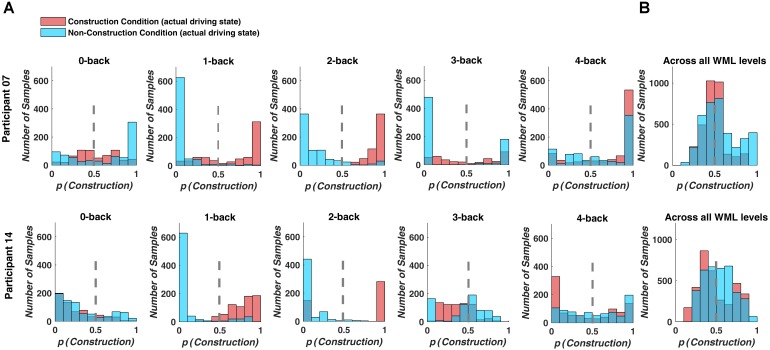
Classifier output predicting driving difficulty for example participants P7 and P14. Colors indicate the actual driving condition and vertical dashed lines indicate the class limit of the logistic regression output. Values larger than 0.5 were assigned to the construction condition. **(A)** For the separate prediction models, most signal samples are predicted correctly at intermediate WML levels (1-back to 3-back level). **(B)** For the combined model, many signal samples are incorrectly classified.

Prediction performance declined, when we used a decoding model that combined the fNIRS data over WML levels to classify driving difficulty. With this procedure, prediction was around chance level with a mean classification accuracy of 46.8% and a mean *F*1-score of 0.419 over all participants (see Table 4). Figure 5 depicts example histograms of the classifier output for two participants. These results show that for seperate models (Figure 5A), prediction of driving difficulty is clearly higher than in the combined model (Figure 5A), suggesting an interaction between brain networks modulated by increasing driving difficulty and brain networks modulated by WML variations. Importantly, this interaction appears to be asymmetric as the reverse was not the case. Unni et al. (2017) demonstrated that WML level can be predicted from fNIRS measurements independent of changes in driving difficulty using data from the same experiment.

**Table 4:** Accuracy and *F*1-score of each classifier across participants for the prediction of driving difficulty across all WML levels.

Participant	P1	P2	P3	P4	P5	P6	P7	P8	P9	P10	P11	P12	P13	P14	P15	Mean
Accuracy(%)	49	54	34	41	61	51	44	41	52	46	42	43	55	42	48	47
*F*1-score	0.44	0.44	0.40	0.42	0.57	0.50	0.42	0.18	0.44	0.31	0.35	0.55	0.52	0.38	0.37	0.42


**FIGURE 6:**
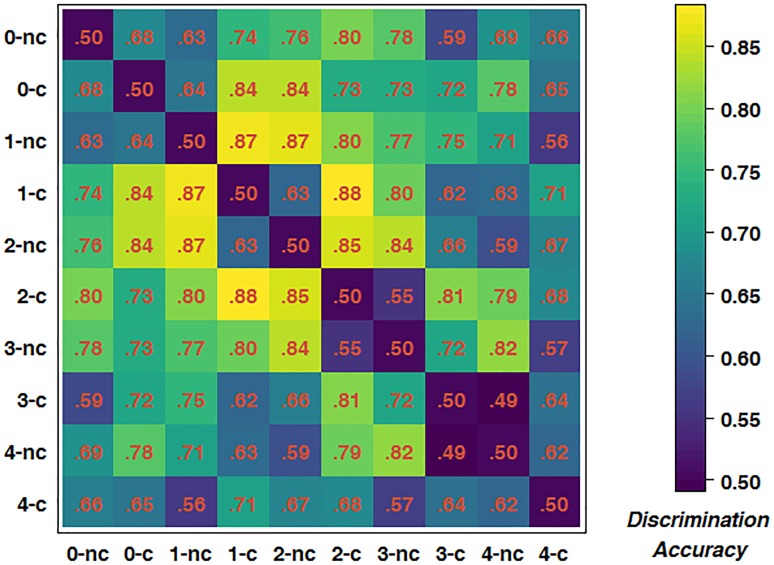
Dissimilarity matrix of predictions of all possible pairings of conditions. c, construction; nc, non-construction; corresponding number indicates WML level.

To further test for an interaction between driving difficulty and WML, we trained classifiers for all possible pairings of experimental conditions to obtain a dissimilarity matrix. As there were five WML levels and each WML level consisted of two different driving difficulty levels, there were ten conditions in total, resulting in 45 pairings. Figure 6 depicts the mean dissimilarity matrix over all participants. Higher discrimination accuracies indicate more reliable changes in brain activations with increasing driving difficulty. In line with the previous analysis, the highest discrimination rates were achieved at intermediate WML levels. This is indicated by accumulation of pairs with higher discrimination rates (depicted by yellow color) in the central areas of the matrix. In addition, a closer analysis of the pattern along the first off-diagonal trace shows an alternating pattern of high and low discrimination accuracies. For example, the 2-back construction brain measurements could be better discriminated from 2-back non-construction than from 3-back non-construction [*t*(14) = 6.311, *p* < 0.001]. This pattern was consistent across other *n*-back levels and summarized in Table 5. The average prediction accuracy of driving difficulty within the same WML level was 75.0%, whereas the prediction accuracy of driving difficulty for adjacent WML levels was 57.8%, with this difference being significant [*t*(14) = 5.854, *p* < 0.001]. This shows that the driving difficulty became less discriminable by fNIRS data once the WML was increased slightly in the non-construction condition, a pattern that is expected when we assume interactions of driving difficulty with varying WML at the brain level.

**Table 5:** Comparison of mean accuracy for prediction of driving difficulty between predictions within WML levels and adjacent WML levels.

	0c		1c		2c		3c		4c		Mean	
**Discrimination of driving difficulty**	**0nc**	**1nc**	**1nc**	**2nc**	**2nc**	**3nc**	**3nc**	**4nc**	**4nc**	
Within WML level	0.68		0.87		0.85		0.72		0.62	0.75
Adjacent WML level		0.64		0.63		0.55		0.49			0.58

Comparison	*t*(14) = 0.601 *p* = 0.558		*t*(14) = 5.913 *p* < 0.001		*t*(14) = 6.311 *p* < 0.001		*t*(14) = 2.736 *p* = 0.016			*t*(14) = 5.854 *p* < 0.001	


#### Localization of Predictive Brain Areas

**FIGURE 7:**
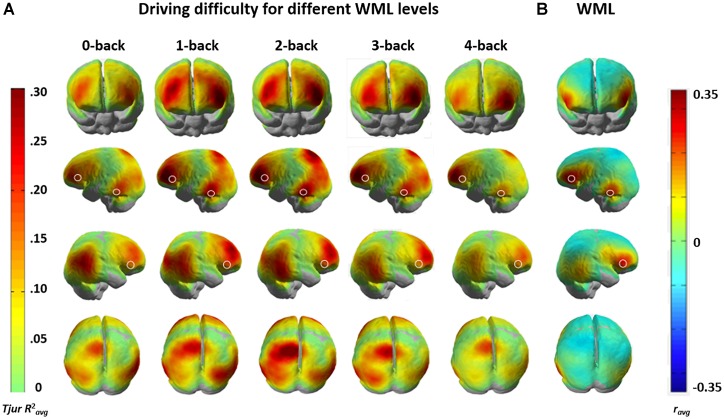
**(A)** Weighted mean of channel-wise predictivity (*Tjur R*^2^*_avg_*) for driving difficulty at the different WML levels. **(B)** Weighted averaged group-level univariate correlation (*r*_avg_) HbR brain maps showing brain areas sensitive to changes in WML independent of driving difficulty. White shapes mark WML prediction maxima in all maps. Data for the two analyses were recorded in the same session with concurrent manipulation of driving difficulty and WML.

To gain further insights into the functional anatomy Kindly confirm whether the heading levels have been correctly identified.of brain areas associated with increased driving difficulty and their modulation by WML level variation, we calculated channel-wise univariate logistic regressions of HbR levels between the construction and non-construction conditions for each participant and each *n*-back level. Figure 7A shows the group-level brain maps depicting classification separability, derived as the weighted averaged channel-wise *Tjur R*^2^ coefficients (*Tjur R*^2^*_avg_*) from the univariate logistic ridge regression model. The maps show that predictivity of fNIRS activation in the lateral dorsal frontal and parietal areas increases up to the 2-back WML level, while the predictivity of fNIRS activation decreases at higher WML levels (i.e., the 3-back and especially the 4-back levels). This follows the pattern of discriminability variation in the multivariate analysis. These results indicate that the loci of interaction between WML and driving difficulty are in the bilateral dorsal frontal (putative BA 46), bilateral inferior parietal (putative BA 39), and left superior parietal (putative BA 7) areas.

We compared the brain maps to the results from Unni et al. (2017) depicted in Figure 7B, where the same fNIRS data was used to predict WML levels independent of driving difficulty (average correlation between predicted and induced WML *r* = 0.61). The comparison of the anatomical locations of predictive maxima for WML predictions in Figure 7B (marked by white shapes) to Figure 7A suggests only partial overlap between the brain resources predictive to the different task demands. Variation of WML level was best predicted in bilateral inferior frontal gyrus (IFG; putative BA 45), an area more posterior to the lateral dorsal frontal areas (putative BA 46) predictive for driving difficulty. An occipito-temporal predictive region (putative BA 21) overlapped between WML and driving difficulty predictors but appeared more left lateralized in WML prediction, which has a stronger language component. The bilateral inferior and left superior parietal areas (putative BA 39 and BA 7, respectively) which showed increased predictivity to driving difficulty seems to show reduced correlations in the WML level predictions (see Supplementary Figure S1 for an annotation of putative Brodmann areas). This suggests that these areas are unique to the prediction of driving difficulty, likely involved in visuomotor attention (Jovicich et al., 2001; Caplan et al., 2006) such as vigilance and tracking of moving objects (Culham et al., 1998), but nevertheless their predictivity depends on WML level.

We visualized the averaged brain map on the MNI 152 brain in the Neurosynth^1^ and used MRIcron^2^ to determine MNI co-ordinates and the corresponding Brodmann areas for the brain areas depicting increased predictive discriminability of the driving difficulty. Table 6 lists the brain areas and their corresponding MNI-co-ordinates of the predictive maxima of the driving difficulty and the WML levels.

**Table 6:** Brain areas showing predictive maxima of the driving difficulty and WML levels and their corresponding MNI co-ordinates.

Brain areas	Putative Brodmann Area (BA)	*X*	*Y*	*Z*
**For driving difficulty**				
Right dorsal frontal	46	38	52	30
Left dorsal frontal	46	-38	50	30
Right inferior parietal	39	44	-76	32
Left inferior parietal	39	-52	-66	18
Left superior parietal	7	-18	-64	70
Left occipito-temporal	21	-68	-38	-4
Right occipito-temporal	21	68	38	4
**For WML levels**				
Right inferior frontal gyrus	45	52	36	20
Left inferior frontal gyrus	45	-52	38	20
Left occipito-temporal	21	-68	-38	-4


## Discussion

In this driving simulator study, we varied visuospatial attention demands by changing the lane widths, thus manipulating driving difficulty while participants performed a modified *n*-back WML speed regulation task. Using almost whole-head fNIRS brain activation measurements, we were able to predict the driving difficulty using a decoding model for each WML level separately. However, the predictions of driving difficulty degraded significantly when we tried to predict driving difficulty using a decoding model which combined fNIRS data over all WML levels.

In order to investigate possible interactions between visuospatial attention and WML, there were two experimental manipulations. To induce different demands in visuospatial attention, participants drove half of the time through a construction site with reduced lane-widths, increasing driving difficulty. At the same time, participants performed a modified *n*-back speed regulation task (0-back to 4-back) resulting in five different levels of WML. Our goal was to predict the driver’s current driving difficulty from almost whole-head fNIRS brain activation measurements using a multivariate, cross-validated logistic ridge regression model. As we were interested in understanding if there exists an interaction between visuospatial attention and WML on a brain level, we predicted driving difficulty with a decoding model which used fNIRS data separately for each WML level and with the same decoding model using fNIRS data combined over all WML levels to compare the decoding accuracies between the models. Our rationale was that if visuospatial attention and working memory had independent underlying brain processes, it should be possible to predict driving difficulty in a combined model across all WML levels. However, this was not the case. In fact, prediction accuracy for driving difficulty across all WML levels was at chance level. Yet, model accuracy improved when the prediction of driving difficulty was calculated separately for each WML level (mean accuracy = 75.0% over all WML levels). Further, there was a significant effect of the WML level on the prediction of driving difficulty.

Thus, we draw two conclusions. First, as driving difficulty could be predicted separately for each WML level, changes in driving difficulty lead to changes in neural correlates detectable by fNIRS. This means that the separate models were able to identify neural correlates specific to changes in driving difficulty for each WML level. Second, the chance level accuracy achieved while predicting driving difficulty in the combined model across different WML levels suggests that no neural correlates measurable with fNIRS changed with driving difficulty across different WML levels. This means, the changes in activation patterns due to changes in driving difficulty depended on the driver’s current WML level. The interaction of the underlying brain processes is further supported by the additional comparisons of all possible combinations of predictions of driving difficulty separately across different WML levels. We showed that the construction condition could be better predicted when discriminated against the non-construction condition at the same WML level than when discriminated against a non-construction condition at the successive WML level. This suggests that an increase in WML recruits a neural network which reduces the discriminability of different levels of driving difficulty.

As fNIRS has good spatial resolution, it allowed us to determine brain areas predictive for visuospatial attention and to study a possible effect of WML on these brain areas. In order to identify potential brain areas associated with increased driving difficulty, we calculated group-level brain maps using univariate channel-wise logistic regression analysis to predict driving difficulty for each WML level. This analysis revealed the bilateral dorsal frontal (putative BA 46), bilateral inferior parietal (putative BA 39), and left superior parietal (putative BA 7) areas to be most sensitive to changes in driving difficulty. Nevertheless, these discriminative patterns diminished at higher WML levels indicating an interaction between visuospatial demands and WML levels.

The bilateral dorsal frontal areas (putative BA 46) are known to be involved in executive control of behavior (Kübler et al., 2006). In contrast, the bilateral inferior parietal (putative BA 39) and left superior parietal (putative BA 7) areas have been associated with visuomotor integration, spatial perception and orientation as well as in visual motion analysis (Andersen, 2011) and visuomotor attention (Jovicich et al., 2001; Caplan et al., 2006) such as vigilance and tracking of moving objects (Culham et al., 1998). These areas play an important role in driving, especially with increased driving difficulty as in this study while driving through a construction site with reduced lane widths.

We proceeded to compare the brain areas predictive to driving difficulty to those areas predictive to WML independent of driving difficulty, previously shown by Unni et al. (2017) using the same data. The comparison of the anatomical locations of predictive maxima for WML predictions revealed only partial overlap between the brain resources predictive to the different task demands. Variations in WML levels was best predicted in bilateral inferior frontal gyrus (IFG, putative BA 45), which was further posterior to the lateral dorsal frontal areas (putative BA 46) predictive for driving difficulty. An interesting point to note was that the bilateral inferior and left superior parietal areas (putative BA 39 and BA 7, respectively), which showed increased predictivity to driving difficulty, showed negative correlations in the WML level predictions independent of driving difficulty. This could indicate that the two tasks interact at a common, task unspecific cognitive resource at the brain level. The changing pattern of driving difficulty related brain areas across WML levels could indicate potential changes in the multitasking strategy with level of WML demand.

The task interactions at brain level could be explained on the basis of the Multiple Resource Theory (Wickens, 2002) where an executive control system adjusts and allocates resources between the two tasks. The bilateral dorsal frontal areas could potentially represent the executive control system. From the predictivity patterns of the brain maps, we observed that these areas show increased predictivity to driving difficulty up to the 3-back WML level suggesting an increase in the difference in effort by the participants for driving difficulty. The increased cognitive resources allocated by the executive control to the WML task rather than for increased visuospatial attention may have reduced the predictivity pattern in the parietal areas representing visuomotor co-ordination. It has been shown in a driving simulator study that participants can strategically prioritize among subtasks and adapt effort and driving behavior accordingly (Cnossen et al., 2000).

In our study, prediction accuracies and *F*1-scores derived from fNIRS brain activation measurements decreased for 3- and 4-back WML levels. Participants might have reached their maximum capacity at 3-back or 4-back WML levels. According to multiple theories (Kahneman, 1973; Tombu and Jolicoeur, 2003; Wickens et al., 2015), once the maximum resource capacity is reached, limited resources are distributed across subtasks. This would suggest that there were only limited resources available for visuospatial attention needed for increased driving difficulty in the higher WML levels. This can explain the drop in task performance, the decrease in prediction accuracies and *F*1-scores, as well as the decreased predictivity of localized brain areas associated with increased driving difficulty for high WML levels.

The notion of a competition of cognitive resources available for the two tasks was further supported by the analysis of the behavioral data. Participants made more errors in the working memory task with increased driving difficulty and had to make more steering adjustments (indicated by higher steering reversal rates) with increased WML levels. Hence, an increase in cognitive demands for one domain led to a decrease in performance associated with the other cognitive domain. These results are in line with Salvucci and Beltowska (2008) who observed that increasing working memory demand of a concurrent task substantially reduced driving performance with respect to lateral control and brake response. Further, this task interference became larger at high WML. Specifically, at high WML levels (3- and 4-back), increased driving difficulty led to a much larger drop in performance in the working memory task, as compared to low and intermediate WML levels (0-back to 2-back) at which the effect of increased driving difficulty on the working memory task performance was substantially smaller.

There are some limitations in this study that need to be addressed. First, our sample population was low. Second, the working memory task used is novel and other than traditional memory span task used in driving research, where digits are presented auditory (e.g., Mehler et al., 2012), the presentation of stimuli in this task was visually, at a lower frequency, which added an additional encoding and retention component to the task. Future studies using the same paradigm should also consider that a participant needs to pass *n*-number of speed signs to reach the corresponding *n*-back WML level and might therefore want to include more speed signs for higher *n*-back levels. Third, the construction condition is not well validated. For example, driving through a construction site is associated with increased workload (Shakouri et al., 2018), even if the lane width is not reduced (Vrieling et al., 2014). For example, the construction condition had different lane markings than the non-construction condition, which can influence driving behavior (Davidse et al., 2004; Charlton et al., 2018). Further, pylons marked the beginning of the construction sites in this experiment that could possibly affect the preferred driving speed in construction sites (Blackman et al., 2014; Steinbakk et al., 2017). In general, rich driving environments increase visual demands and uncertainty in the driver (Kujala et al., 2016), which might have made it more difficult for the driver to detect and encode speed signs in the construction condition necessary for the WML task. Thus, increased effort in scanning for speed signs in the construction condition could have altered lane-keeping. We also have to point out that participants received feedback for the working memory task only, possibly shifting the focus toward this task, whereas in real driving, lane keeping would have been prioritized over speed regulation. To assure participants had the effective WML level as intended, we have excluded time segments, in which participants didn’t reach their target speed.

Our results could potentially have practical implications in the field of brain-based adaptive driver state assessment. Assessment of a driver’s cognitive state has the goal to detect when the driver’s workload is too high to keep up with the demands of operating a vehicle safely (Aghaei et al., 2016). In such situations, a driver assistance system could provide feedback to the driver (Feng and Donmez, 2013). For example, the use of a haptic steering wheel providing haptic feedback to the driver for ideal steering movements helped to decrease driving difficulty (Steele and Gillespie, 2001). Alternatively, adaptive automation systems have the goal to detect the driver’s current cognitive demands and to adjust the level of automation accordingly (Parasuraman et al., 2000). Our results illustrate the challenge to disentangle different types of workloads calling for new methods in workload assessment for an accurate assessment of cognitive demands in applied multiple task settings.

## Conclusion

Our study indicates brain level interactions between visuospatial attentional demands and WML while driving using fNIRS brain activation measurements. As an explanation for the dependency of those two different cognitive demands, we proposed that once maximum capacity is reached, the two tasks must compete for available resources. Further, there could be an interaction at a common, task unspecific cognitive resource at the brain level. The interaction of those different driving relevant tasks constitutes a challenge in brain-based driver state assessment for adaptive automation systems. Future studies should investigate how different subtasks in driving influence each other and how they could be assessed independently. This could eventually lead to more specific support for the driver in operating the car safely.

## Ethics Statement

The experiments of this study were carried out in accordance with the recommendations of the guidelines of the German Aerospace Center and approved by the ethics committee of the Carl von Ossietzky University, Oldenburg, Germany. All subjects gave written informed consent in accordance with the Declaration of Helsinki.

## Author Contributions

AU, KI, MJ, and JR planned the research. AU and KI done data collection. JS, AU, and JR carried out data analysis. JS, AU, KI, MJ, and JR prepared the manuscript.

## Conflict of Interest Statement

The authors declare that the research was conducted in the absence of any commercial or financial relationships that could be construed as a potential conflict of interest.

## Footnotes

1 http://neurosynth.org

2 https://www.nitrc.org/projects/mricron
