# Supplementary material for: Demonstrating Brain-Level Interactions Between Visuospatial Attentional Demands and Working Memory Load While Driving Using Functional Near-Infrared Spectroscopy
Source: Front Hum Neurosci. 2019 Jan 23;12:542. doi: 10.3389/fnhum.2018.00542 (PMC6351455; doi:10.3389/fnhum.2018.00542)
Supplement: Supplementary file 1 [file Data_Sheet_1.docx]

**Table S1.** *Differences in standard deviation of lane position (in meters) between driving difficulty conditions (construction – non-construction) calculated via paired-sample t-test. Lane changes were excluded from the analysis.*

|  | 0-back | | 1-back | 2-back | 3-back | 4-back | Mean |
| --- | --- | --- | --- | --- | --- | --- | --- |
| Construction | | .255 | .276 | .264 | .258 | .241 | .259 |
| non-construction | | .262 | .292 | .257 | .302 | .267 | .276 |
| t_construction−non-construction_  Significance test | | *t*(13)=-0.748  *p*=..468 | *t*(13)=-1.302  *p*=.216 | *t*(13)=0.513  *p*=.617 | *t*(13)=-4.666  *p*<.001 | *t*(13)=-1.856  *p*=.086 | *t*(13)=-2.803  *p*=.015 |
| bonferroni corrected p*-value | | *p*=*1.00 | *p*=*1.00 | *p*=*1.00 | *p**<.01 | *p*=*.431 | *p**=.075 |
