# Supplementary figures and images for: Demonstrating Brain-Level Interactions Between Visuospatial Attentional Demands and Working Memory Load While Driving Using Functional Near-Infrared Spectroscopy
Source: Front Hum Neurosci. 2019 Jan 23;12:542. doi: 10.3389/fnhum.2018.00542 (PMC6351455; doi:10.3389/fnhum.2018.00542)

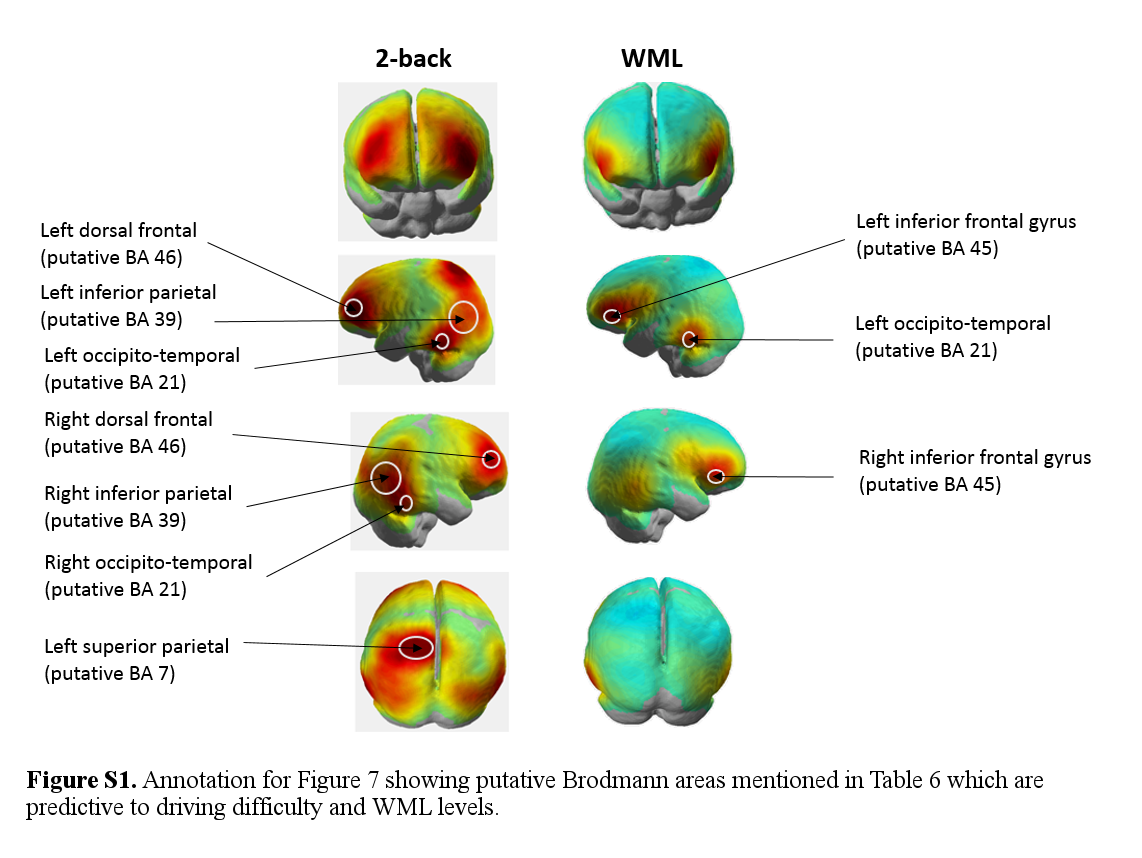

Supplement: Supplementary file 2 [file Image_1.png]
